# Genome sequences of eight *Fusobacterium watanabei* sp. isolates

**DOI:** 10.1128/mra.00132-25

**Published:** 2025-07-28

**Authors:** Martha A. Zepeda-Rivera, Falk Ponath, Kaitlyn N. Lewis, Rutika P. Gavate, Floyd E. Dewhirst, Junko Tomida, Yoshiaki Kawamura, Kaori Tanaka, Susan Bullman, Christopher D. Johnston

**Affiliations:** 1 Genomic Medicine, MD Anderson Cancer Center, Houston, Texas, USA; 2 Immunology, James P. Allison Institute, MD Anderson Cancer Center, Houston, Texas, USA; 3 ADA Forsyth Institute, Cambridge, Massachusetts, USA; 4 Department of Oral Medicine, Infection and Immunity, Harvard School of Dental Medicine, Boston, Massachusetts, USA; 5 Department of Microbiology, School of Pharmacy, Aichi Gakuin University, Nagoya, Aichi, Japan; 6 Division of Anaerobe Research, Life Science Research Center, Gifu University, Yanagido, Gifu, Japan; University of Maryland School of Medicine, Baltimore, Maryland, USA

**Keywords:** *Fusobacterium*, Illumina sequencing

## Abstract

We report draft genome sequences of eight *Fusobacterium watanabei* clinical isolates, ranging from 1.95 to 2.09 Mbp. Analysis against the Genome Taxonomy Database indicates that *F. watanabei* genomes are part of the “*Fusobacterium nucleatum_J*” group.

## ANNOUNCEMENT

*Fusobacterium watanabei* was previously isolated from clinical samples in Japan and described by Tomida et al. (1) as a novel species closely related to the *Fusobacterium nucleatum sensu lato* subspecies. Here, we report and release the assembled genome sequences for these eight *F. watanabei* isolates (Table 1).

**TABLE 1:** *Fusobacterium watanabei* genomes**^*a*^**

Genome name	Isolate provenance	Sample source	Size (bp)	GC (%)	No. of contigs	No. of reads	*N*_50_	Assembly accession	Raw read accession
PAGU 1795	Hiroshima, Japan	Ascitic fluid	2,015,742	26.89	48	1,310,315	177,963	GCA_965217525	ERR14995307
PAGU 1796	Aomori, Japan	Ascitic fluid	1,951,877	26.86	44	1,292,750	144,056	GCA_965217535	ERR14995308
PAGU 1797	Kyoto, Japan	Pleural diffusion	2,005,769	26.98	34	1,235,122	278,257	GCA_965217465	ERR14995309
PAGU 1798	Osaka, Japan	Pus (neck)	2,012,989	26.85	44	1,422,737	317,217	GCA_965217495	ERR14995310
PAGU 1799	Kyoto, Japan	Pus (oral cavity)	2,087,850	26.82	53	1,421,568	217,125	GCA_965217515	ERR14995312
PAGU 1800	Tokyo, Japan	Pleural effusion	2,009,578	26.90	92	1,342,333	120,039	GCA_965217485	ERR14995313
PAGU 1801	Kyoto, Japan	Pleural effusion	2,004,759	26.99	41	1,461,651	278,257	GCA_965217505	ERR14995314
PAGU 1802	Kyoto, Japan	Intraperitoneal exudate	2,005,282	26.98	42	1,595,261	159,188	GCA_965217475	ERR14995315

^*a*^ Table shows each *Fusobacterium watanabei* isolate name, with assembly metrics and assembly and raw read accession numbers.

The 16S rRNA gene and partial sequences of four marker genes for the PAGU 1796/CCUG 74246 (type strain) were available at the National Center for Biotechnology Information with sequence data available in the Short Read Archive (DRA009376 and DRA009930). More recently, the type strain genome has been made publicly available (PRJEB85514, GCA_965119615.1). Here, we release assembled genome sequences for eight *F. watanabei* isolates. Strains were anaerobically grown on blood agar plates (Remel, CDC formulation, R01036) at 37°C (Baker Concept 1000; N_2_:H_2_:CO_2_ 90:5:5). Colonies appearing by 48 h were used for genomic DNA isolation using a modified protocol with the Monarch Spin gDNA Extraction Kit (New England Biolabs, Ipswich, MA, USA). Briefly, the resuspended bacterial pellet and lysis buffer were combined and transferred to an MP Lysing Matrix B tube (MP Biomedicals, Santa Ana, CA, USA), followed by mechanical disruption using the MP Biomedicals Fastprep-24 Bead Beater (MP Biomedicals, Santa Ana, CA, USA) at 4.0 m/s for 20 s. Subsequent steps followed the standard kit protocol. Illumina sequencing libraries were prepared using the tagmentation-based and PCR-based Illumina DNA Prep Kit (Illumina, San Diego, CA, USA) and custom IDT 10 bp unique dual indices (Integrated DNA Technologies, Inc., Coralville, IA, USA) with a target insert size of 280 bp. Illumina sequencing was performed on an Illumina Novaseq X Plus sequencer in one or more multiplexed shared-flow-cell runs, producing 2 × 151 bp paired-end reads. Demultiplexing, quality control, and adapter trimming were performed with bcl-convert (v4.2.4) under default parameters. Genome assembly was performed with Unicycler (2) (v0.5.0) under default parameters. Resulting genome assemblies indicate a genome size ranging from 1.95 to 2.09 Mbp (average of 2.01 Mbp) with GC content ranging from 26.82% to 26.99% (average of 26.92%) (Table 1).

**Fig 1:**
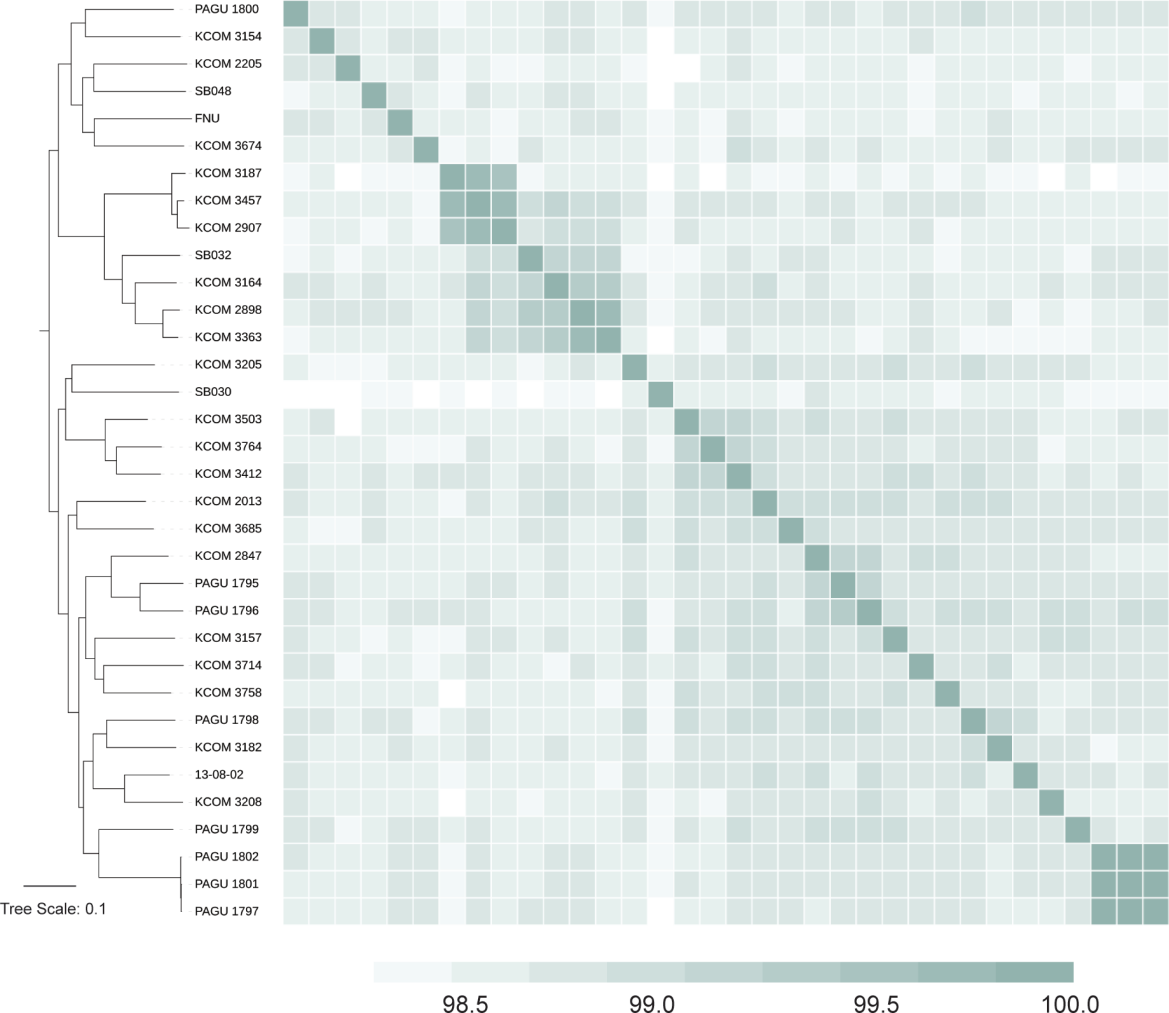
Average nucleotide identity between *Fusobacterium watanabei* and closest publicly available relatives. Figure displays (left) a kSNP4 (8) whole-genome reference-free phylogenetic tree (kmer = 23, FCK = 0.714) of *Fusobacterium watanabei* genomes, including the previously published “*Fusobacterium nucleatum_J”* group genomes with (right) corresponding FastANI comparisons. The color of each individual box represents the fastANI value between a genome pair, ranging from 98.0% (white) to 100% (dark green). ANI heatmap generated with pheatmap R package, RStudio version 2024.12.0+467. Tree was visualized using iTOL (9) (v7.2).

Taxonomic identification of these *F. watanabei* assembled genomes against the Genome Taxonomy Database using GTDB-tk (3) (v2.3.2) indicated that *F. watanabei* isolates belong to the “*Fusobacterium nucleatum_J*” group, which also encapsulates the strain 13-08-02, strain “*Fusobacterium nucleatum* subsp. *unique* (FNU)” previously described in Ma et al. (4), and members of the group we recently described as “*Fusobacterium nucleatum* subsp. *animalis* clade 1 (*Fna* C1)” (5). Pairwise average nucleotide identity values between these genomes were calculated using FastANI (6) (v0.1.3) (Fig. 1). These results supported a previous observation based on single marker gene analysis that “*Fusobacterium watanabei*” and “*Fna* C1” may represent the same lineage (Øyvind Kommedal, personal communication, October 2024). With the public release of the assembled genomes here, *F. watanabei*, an already recognized species name under the List of Prokaryotic names with Standing in Nomenclature (7), can be appropriately applied to genomes within “*F. nucleatum_J*” in GTDB. This increases the number of representative genomes for *Fusobacterium watanabei*, currently the closest known phylogenetic clade to the colorectal cancer-associated *Fusobacterium animalis* (5).

## Data Availability

Sequencing read data and whole-genome assemblies for PAGU 1795, PAGU 1796, PAGU 1797, PAGU 1798, PAGU 1799, PAGU 1800, PAGU 1801, and PAGU 1802 are available in ENA under the study number PRJEB87340 with the following accessions: GCA_965217525 (PAGU 1795), GCA_965217535 (PAGU 1796), GCA_965217465 (PAGU 1797), GCA_965217495 (PAGU 1798), GCA_965217515 (PAGU 1799), GCA_965217485 (PAGU 1800), GCA_965217505 (PAGU 1801), and GCA_965217475 (PAGU 1802).
